# Genetic Variants in the Fat Mass and Obesity‐Associated Gene and Risk of Obesity/Overweight in Children and Adolescents: A Systematic Review and Meta‐Analysis

**DOI:** 10.1002/edm2.510

**Published:** 2024-07-07

**Authors:** Maryam Eghbali, Azadeh Mottaghi, Sara Taghizadeh, Sara Cheraghi

**Affiliations:** ^1^ Endocrine Research Center, Institute of Endocrinology and Metabolism Iran University of Medical Sciences Tehran Iran; ^2^ Research Center for Prevention of Cardiovascular Diseases, Endocrinology & Metabolism, Institute of Endocrinology Metabolism Iran University of Medical Sciences Tehran Iran; ^3^ Translational Ophthalmology Research Center Tehran University of Medical Sciences Tehran Iran

**Keywords:** adolescent, children, fat mass and obesity‐associated gene, obesity, overweight, single nucleotide polymorphism

## Abstract

**Objective:**

The variations in the single‐nucleotide polymorphisms (SNPs) of the fat mass and obesity (FTO)‐associated gene have been linked to being overweight or obese in children. In this research a thorough examination was performed to elucidate the connection between various FTO gene SNPs and overweight or obesity in children and adolescents.

**Method:**

We searched PubMed, Google scholar, Web of Science and Scopus until January 2024 to find studies that investigate the association between different SNPs of FTO gene and the risk of overweight/obesity in children and adolescents. After filtering the relevant studies, meta‐analysis was used to quantify the association of FTO gene SNPs within different genetic inheritance models.

**Results:**

We have identified 32 eligible studies with 14,930 obese/overweight cases and 24,765 healthy controls. Our recessive model showed a significant association with rs9939609 (OR: 1.56, 95% CI: 1.20; 2.02, *p* < 0.01) and rs1421085 (OR: 1.77, 95% CI: 1.14; 2.75, *p* < 0.01). Besides, in the homozygote model, rs1421085 showed the highest association (OR: 2.32, 95% CI: 1.38; 3.89, *p* < 0.01) with the risk of obesity in a population of children and adolescents. Moreover, there are other SNPs of FTO genes, such as rs9921255, rs9928094 and rs9930333, which showed a positive association with obesity and overweight. However, their effects were evaluated in very few numbers of studies.

**Conclusion:**

In this study, we have found that the FTO rs9939609 and rs1421085 are associated to an increased risk of obesity among children and adolescents. Besides, the findings of this study further reaffirmed the established link between rs9939609 and obesity in children and adolescents.

## Graphical abstract

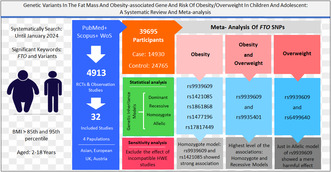

## 1 Introduction

The rising problem of obesity is impacting individuals of all ages [1, 2]. Recent reports from the CDC (Center for Disease Control and Prevention), covering the period from 2017 to 2020, reveal that more than 19% of children and adolescents are facing obesity. Additionally, the WHO estimates that an almost 39 million children under the age of five are either overweight or obese [3, 4]. This alarming trend is especially prominent in Middle Eastern countries, where obesity rates among children and adolescents are on the rise [5].

Obesity is characterised by the accumulation of excess body fat, which can lead to various health problems. Body mass index (BMI) of more than 85th and 95th percentile is categorised as overweight and obesity in children aged 2 years and older, respectively [6, 7, 8]. Childhood obesity can raise the likelihood of developing chronic diseases later in life [9]. Obesity is linked to numerous disorders including stroke, heart disease, type 2 diabetes, certain cancers, gout, osteoarthritis, gall bladder problems, high cholesterol, metabolic syndrome and sleep apnoea [10, 11].

Obesity is affected by a combination of genetic and environmental elements. Consuming high‐calorie diets and leading a sedentary lifestyle are the key environmental factors contributing to obesity. Furthermore, research through genome‐wide association studies (GWAS) has identified specific genes linked to obesity. In 2007, the fat mass and obesity (FTO)‐associated gene was first identified during a GWAS study focusing on diabetes as a susceptible obesity gene [12]. At the same period, two independent teams also reported that the FTO gene was associated with obesity [13, 14]. Studies on different ethnicities have demonstrated that genetic variations in FTO gene influence the development of obesity [15]. FTO with 9 exons is a large gene that covers 400 kb on chromosome 16q12.2 and is highly expressed in various tissues including the brain and hypothalamus [12, 16]. Most human tissue contains FTO mRNA expression, the highest expression level of FTO was detected in the arcuate nucleus (ARC) of the hypothalamus, where it encodes nucleic acid demethylase. The demethylase plays an important role in regulating nucleic acid demethylation, energy homeostasis and lipolysis [17, 18]. High levels of FTO expression are associated with adipogenesis and the production of white adipocytes [19]. FTO is propounding in the obesity studies. The association of FTO single‐nucleotide polymorphisms (SNPs) and BMI [20] and metabolic indicator such as insulin resistance, glucose, triglycerides and cholesterol levels has been shown by the GWAS studies [21]. Various studies conducted on different populations have revealed different levels of association between FTO gene SNPs and obesity. A recent meta‐analysis study has just demonstrated the association of rs9939609 in Western and Asian countries. However, there are conflicting findings regarding the role of FTO in the incidence of obesity in several studies [22, 23, 24]. Although some studies confirmed the significant association between FTO and obesity, others did not endorse these results [25, 26, 27]. In line with this controversy one study showed that fat mass in the FTO deficient mice is equal to the wild‐type controls [22]. Furthermore, hyperplasia was detected in the both over expressing mice and FTO‐knockout mice [25, 27].

This comprehensive review will focus on examining the various research studies that have been carried out on the FTO gene in relation to childhood and adolescent obesity and overweight. However, it is notable to mention that, as the association of the rs9939609 variant of FTO gene has been widely studied by previous reviews [28], in the current report there is a particular focus on the other SNPs of FTO genes. The aim is to identify the key genetic variations that play a significant role in these conditions. The insights gained from this review will enhance our understanding of the genetic factors that contribute to obesity and overweight in young individuals.

## 2 Method and Materials

### 2.1 Search Strategy

A systematical search was conducted using PubMed, Google scholar, Scopus and Web of Science until January 2024 to retrieve all relevant for the FTO and variants and any significant keywords. The keywords and search strategy included: “obesity,” “overweight,” “body mass index,” “FTO,” “fat mass and obesity associated,” “children” and “adolescent.” The search was limited to only the English reports. Moreover, additional studies were revealed by a manual search including studies in systematic review and meta‐analysis studies on the subject. The search strategy for each source was provided as Supporting Information. All the phase of this study was in line with recent version PRISMA guideline [29].

### 2.2 Inclusion and Exclusion Criteria

The studies that are included in this systematic and meta‐analysis comprised the following criteria: case–control, cross‐sectional and cohort studies that compare overweight/obese children with the healthy control group. Studies that contained genotype and allele frequency and determining the relation of FTO variants and overweight/obese in children and adolescents. Moreover, our exclusion criteria were comprised of (1) studies with no control group, (2) studies on adults, (3) studies on case group with secondary obesity or metabolic disorder, (4) case reports, animal studies, letters, abstract (without sufficient data), reviews and (5) studies with overlap genotype data of previous publications by same authors or groups. It should be noted that the studies in which Hardy–Weinberg equilibrium (HWE) was not observed was not excluded. We removed their effect from the final pooled estimate in sensitivity analysis.

### 2.3 Data Extraction

Eligible studies independently were extracted by four investigators (SC, ME and ST). The articles information, sample size, sex, ethnicity, age, BMI, SNPs reference number, genotypes distribution, genotyping methods and HWE states were extracted. The investigators discussed and resolved any disagreements. Sample size, HWE, control group, detection methods and ethnicity have been examined because they can influence the FTO gene polymorphism and overweight/obese risk relationship. Moreover, as different risk alleles were introduced for some SNPs of FTO gene, in order to be more uniform from methodologic perspective, we decided to use the risk alleles introduced in the dbSNP database (www.ncbi.nlm.nih.gov/snp/).

### 2.4 Outcomes

Upon reviewing the included studies, it was observed that the association was reported in three different ways. The majority of studies showed this association only in populations of obese children and/or adolescents. Some studies, on the other hand, demonstrated this association in a combined population of obese and overweight children. Finally, a small number of studies evaluated the association of FTO gene polymorphisms in the overweight children population. Therefore, the outcomes were categorised based on the composition of the samples in the included studies.

### 2.5 Statistical Analysis

For the meta‐ analysis R (version 4.2.1; R Core Team, 2022) was employed for analysis. In this study, we developed five different forms of analysis based on inheritance models. However, it should be noted that, as there are different risk alleles for different SNPs of FTO genes, the format demonstrating each model is symbolic and does not refer to a particular SNP. Also, we considered allele ‘A’ as the risk allele in this symbolic demonstration of the genetic models. Accordingly, the dominant model was defined based on the following format ‘AA + Aa versus aa’. The recessive model was defined as ‘AA versus Aa +aa’. Likewise, we consider two different models which we defined as follows: ‘AA versus aa’ (known as homozygote model) and ‘Aa versus aa’. Finally, we considered the allelic model based on frequency of each allele in the case and control group. To perform the meta‐analysis, we divided our main data set into three groups. In one data set, we only included the studies which reported the effect of each of SNPs in a population of children and/or adolescents with obesity. The second data set only contained data of studies that evaluated the effect of SNPs on mixture of obese and overweight children and/or adolescents. Eventually, the third one comprised of the reports that only included the effect of SNPs on overweight children and/or adolescents. Then we used dichotomous data meta‐analysis using ‘metabin’ command of ‘meta’ package of R [62]. To select which model to report, fixed versus random, we used the value of *I* ^2^. The *I* ^2^ values served as a measure of heterogeneity [63]. The fixed effect model was applied, if *I* ^2^ < 50% and *p* > 0.1 between studies. To measure publication bias in the collected studies funnel plots was created alongside checking Egger's test. It should be noted that due to restrictions exist for conducting such an analysis, we only evaluated the presence of possible publication bias for rs9939609. *p* values < 0.05 were considered statistically significant.

## 3 Results

### 3.1 Description of Studies

**FIGURE 1:**
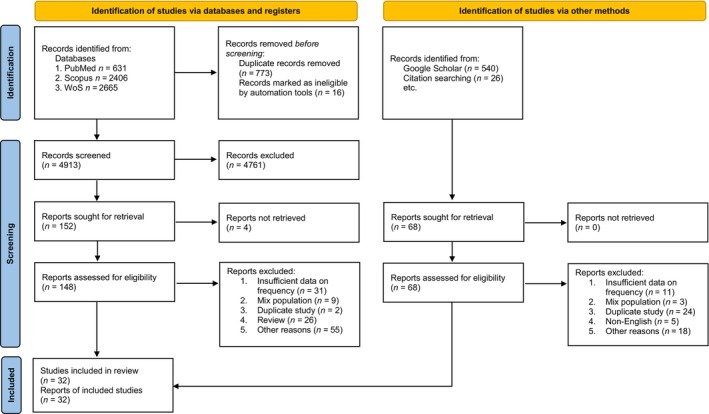
Flowchart of relevant studies' selection process according PRISMA guideline.

In total, 4913 studies were identified by searching the electronic databases. After excluding duplicates and ineligible studies, 32 studies with 14,210 as case and 23,922 as control participants were included in the meta‐analysis (Figure 1). As, in this study there was no specification over particular SNP, we included all the reported SNPs which their effect on risk of obesity and overweight in children and adolescents were evaluated. The SNPs which were reported at least in two different studies were considered for the final meta‐analysis.

### 3.2 Search Results

We identified 20 titles during the initial systematic search (Figure 1). Then 12 additional studies were added after checking reference lists of relevant studies alongside manual search of Google Scholar. A total 32 articles met the inclusion criteria. Out of the 32 studies, nine studies were from China [43, 46, 49, 50, 52, 53, 55, 56, 60], three studies were from Spain [39, 42, 44], two studies were from India [35, 45]. Other studies were once seen in the following countries: Iran [61], Iraq [64], Poland [58], Brazil [54], Italy [51], Mexico [41], Pakistan [48], Portugal [40], Russia [32], Sweden [31], Turkey [37], United Kingdom [36], Czech Republic [59], Germany [30], Austria [34], Chile [47], Egypt [38] and Indonesia [33].

### 3.3 Demographics

An overview of general characteristics of the included studies is displayed in Table S1. The sample size varied from 24 to 1423 in case group and from 45 to 4022 in healthy participants. Overall, there was 14,930 participants in case group and 24,765 in control group. Most of the studies based their inclusion criteria for guidelines, using different cut‐offs such as BMI or percentiles for definition of obesity and overweight among the population of children and/or adolescents.

### 3.4 Effect of Different FTO Gene SNPs on Risk of Obesity

**FIGURE 2:**
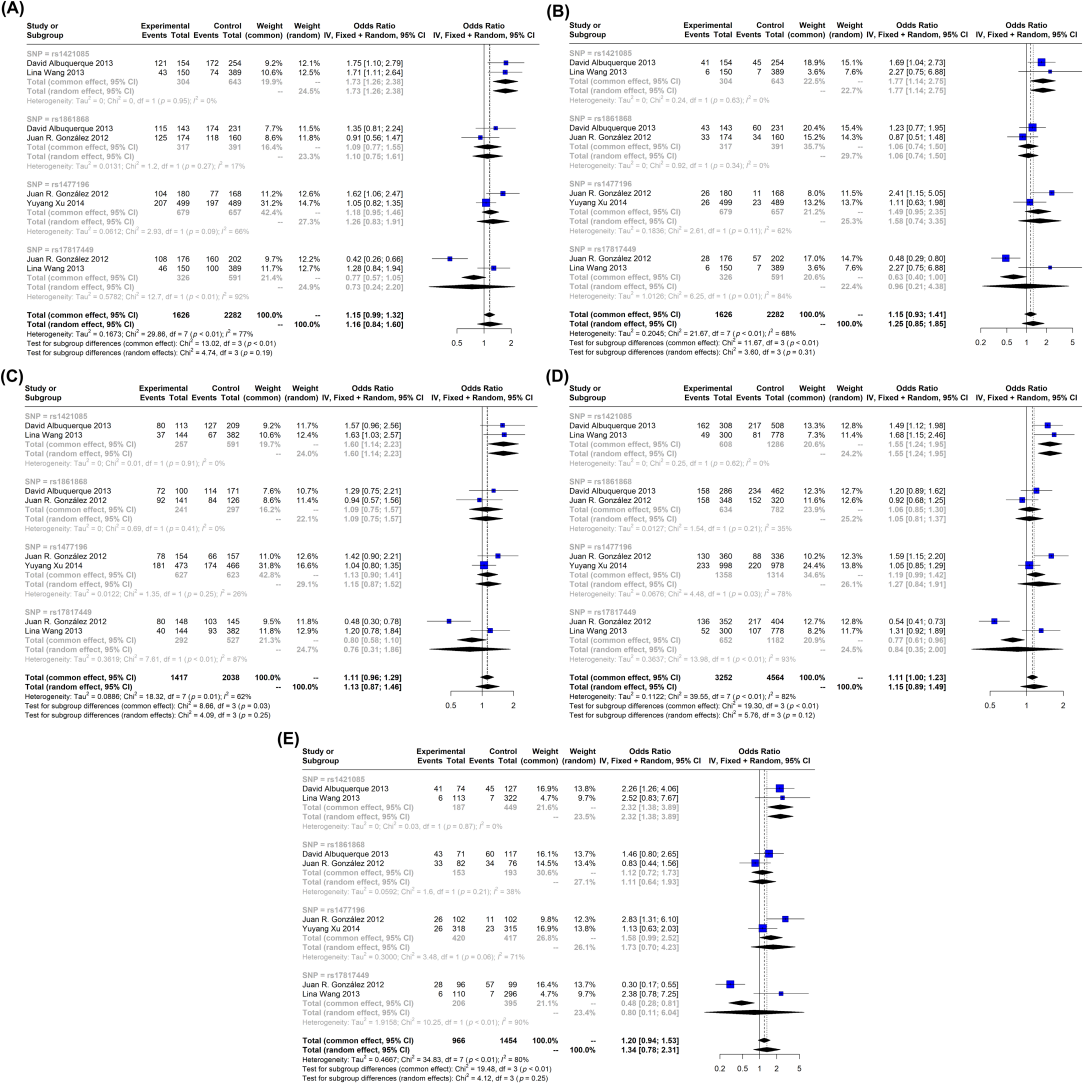
Forest plot of association of fat mass and obesity (FTO) gene polymorphisms with obesity in children and adolescents with obesity. (A) Dominant, (B) recessive, (C) allelic, (D) Aa versus aa and (E) homozygote. The outputs of our analysis for rs9939609 variant are provided in Supporting Information.

Among the evaluated SNP of FTO gene in children and adolescents with obesity, only five SNPs (rs9939609, rs1421085, rs1861868, rs1477196 and rs17817449) were assessed in several studies. Among these five SNPs, two of them, rs9939609 (OR: 1.32, 95% CI: 1.14; 1.54) and rs1421085 (OR: 1.73, 95% CI: 1.26; 2.380), increased the risk of obesity in the dominant model (Figure 2 and Figure S1). The same SNPs showed similar effects in the recessive and allelic models (Figure 2 and Figures S2 and S5). Besides, in homozygote model, rs9939609 and rs1421085 showed strong association with obesity (OR: 1.72, 95% CI: 1.28; 2.31 and OR: 2.32, 95% CI: 1.38; 3.89, respectively). In the other model (Aa vs. aa), rs1421085 showed significant association (Figure 2). Forest plot for all five SNPs are available in the Figures S1–S5.

### 3.5 Effect of Different FTO Gene SNPs on Risk of Obesity and Overweight

**FIGURE 3:**
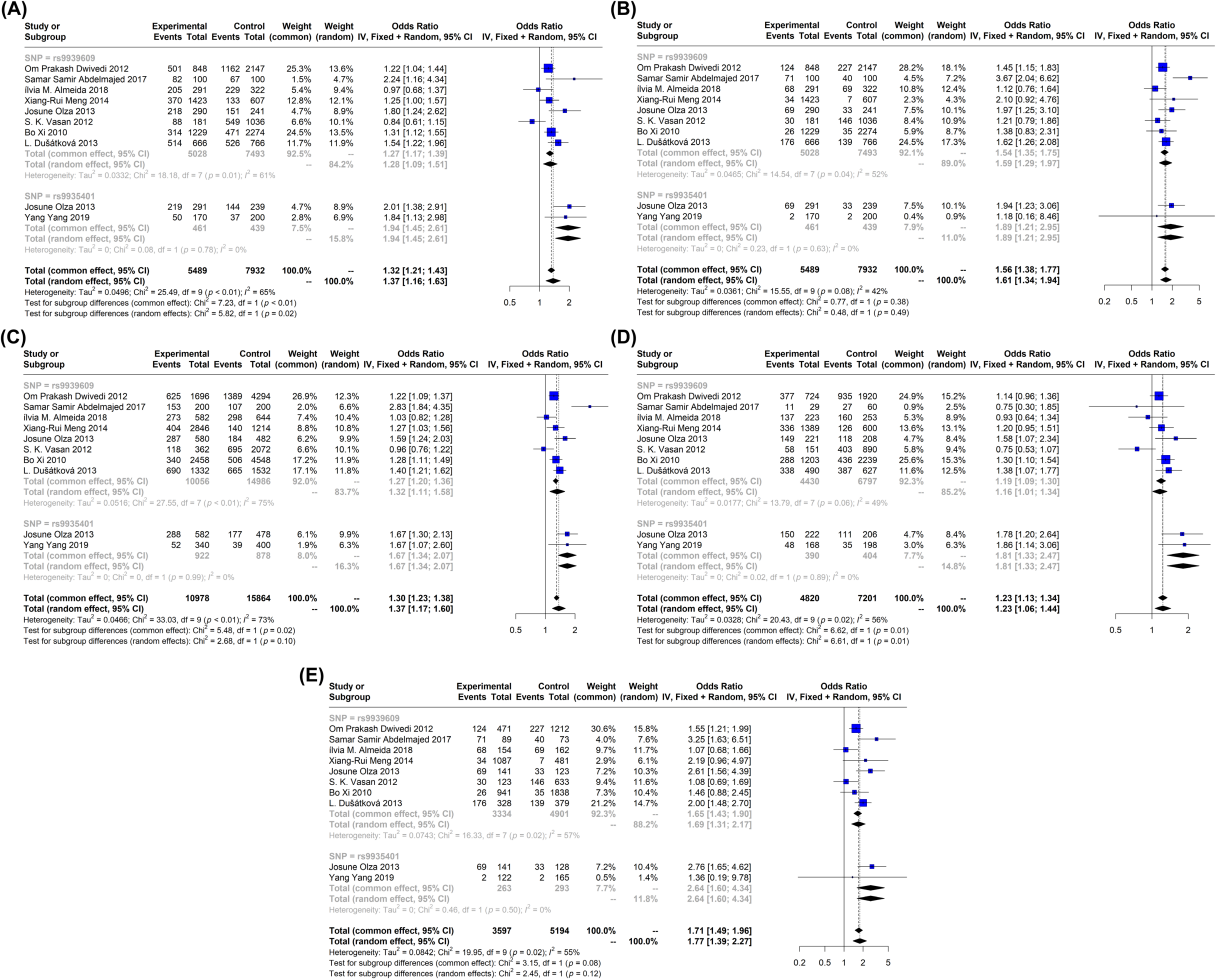
Forest plot of association of fat mass and obesity (FTO) gene polymorphisms with obesity and overweight in children and adolescents. (A) Dominant, (B) recessive, (C) allelic, (D) Aa versus aa and (E) homozygote.

The effect of only two SNPs, rs9939609 and rs9935401, were evaluated on the combined population of obese and overweight children and adolescents in different studies. Both of these SNPs have risen the risk of obesity and overweight in our target population. According to our analysis, the OR of the rs9939609 in dominant and recessive models were 1.28 (95% CI: 1.09; 1.51) and 1.59 (95% CI: 1.29; 1.97), respectively. Regarding the rs9935401, the highest level of the associations was observed in homozygote 1.77 (95% CI: 1.39; 2.27) and recessive 1.56 (95% CI: 1.38; 1.77) models (Figure 3).

### 3.6 Effect of Different FTO Gene SNPs on Risk of Overweight

**FIGURE 4:**
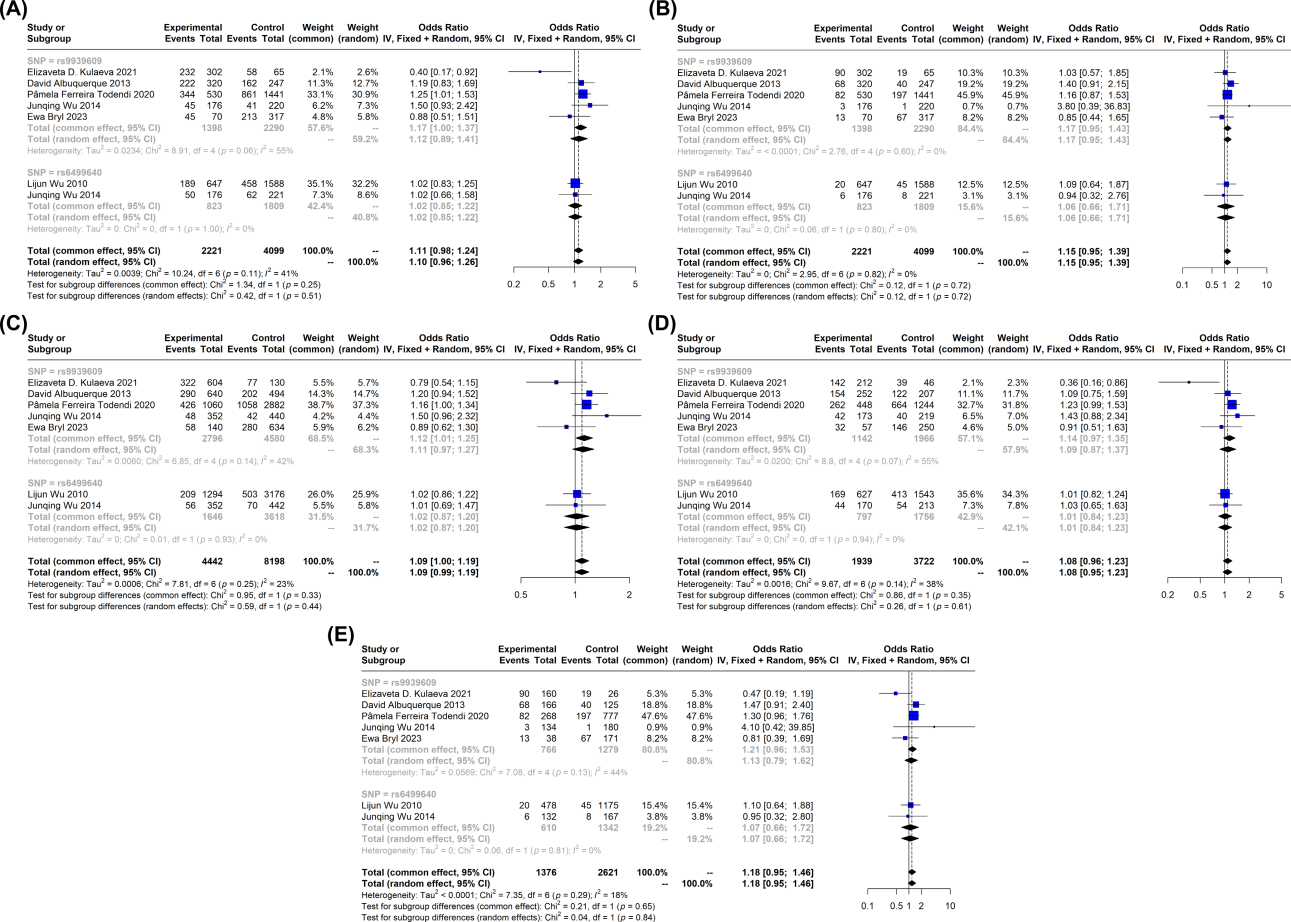
Forest plot of association of fat mass and obesity (FTO) gene polymorphisms with overweight in children and adolescents. (A) Dominant, (B) recessive, (C) allelic, (D) Aa versus aa and (E) homozygote.

The number of studies which assessed the effect of SNPs of FTO genes on risk of overweight were very few. Aside from rs9939609, only one other SNP, rs6499640, was evaluated in different studies. Interestingly none of these two SNPs showed any association with an increased risk of being overweight in children, but in the allelic model of rs9939609 which showed a mere harmful effect (A vs. T) (OR: 1.12, 95% CI: 1.01–1.25) (Figure 4).

### 3.7 Qualitative Assessment

Aside from the SNPs which were included in the meta‐analysis, there were some other SNPs evaluated only in one report. Table 1 summarises the effect of these SNPs in population of children and adolescents. In this regard some reports indicated that rs9921255, rs6499640 and rs72066790 had association with increased risk of obesity. There were two more SNPs (rs9928094 and rs9930333) which showed correlation with high risk of obesity and over weight in a population of Spanish children and adolescents. As these associations are only observed in a single study, further validations are needed to provide a solid picture over the correlation of these SNPs with obesity and overweight.

**TABLE 1:** The characteristics of the included studies.

Author name	Disease phenotype	Population	Sample size case/Control	Country	SNP	Genotypes		Detection method
						Case W/HET/M	Control W/HET/M	
A. Hinney [30]	Severe obesity	Children/Adolescents	487/442	Germany	rs1121980	104/247/133	153/218/71	MT MS
					rs9939973	104/250/130	153/218/71	
					rs7193144	121/241/122	165/211/65	
					rs9940128	106/254/126	153/218/71	
					rs8050136	123/240/123	165/212/65	
					rs9939609	123/241/120	164/214/64	
J. Jacobsson [31]	Overweight	Children/Adolescents	450/512	Sweden	rs9939609	133/206/111	174/244/92	Real‐time
E. D. Kulaeva [32]	Overweight	Children/Adolescents	302/65	Russia	rs9939609	70/142/90	7/39/19	AS‐PCR
S. M. Lubi [33]	Early‐onset obesity	Children	105/107	China	rs9939609	67/32/6	62/37/8	Real‐time
H. Mangge [34]	Obesity	Adolescents	268/103	Austria	rs9939609	75/118/75	31/56/16	Real‐time
O. P. Dwivedi [35]	Obesity/Overweight	Children	896/2230	India	rs9939609	347/377/124	985/935/227	iPLEX assay
					rs8050136	351/397/126	980/944/236	
J. Wardle [36]	Early‐onset obesity	Children	926/4022	United Kingdom	rs9939609	250/389/287	1548/1878/596	Real‐time
N. Çöl [37]	Obesity	Adolescents	100/100	Turkey	rs9939609	32/48/20	41/36/23	SNaPshot
S. S. Abdelmajed [38]	Obesity	Children	100/100	Egypt	rs9939609	18/11/71	33/27/40	RFLP‐PCR
					rs17817449	44/35/21	33/50/17	
D. Albuquerque [39]	Obesity/Overweight	Children	320/256	Portuguese	rs9939609	98/154/68	85/122/40	Real‐time
					rs1421085	98/161/60	82/127/45	
					rs1861868	55/150/89	57/114/60	
M. Almeida [40]	Obesity/Ooerweight	Children	291/322	Portuguese	rs9939609	86/137/68	93/160/69	Real‐time
P. García‐Solís [41]	Obesity	Children	111/343	Mexico	rs9939609	70/30/11	240/93/10	Real‐time
J. R. González [42]	Severe obesity	Children	202/184	Spain	rs2241179	64/90/25	73/75/21	Real‐time
					rs1477199	116/58/8	127/40/3	
					rs7203521	73/85/25	73/79/19	
					rs1861868	49/92/33	42/84/34	
					rs10852521	49/89/39	85/98/26	
					rs1477196	76/78/26	91/66/11	
					rs17817449	68/80/28	42/103/57	
					rs9939609	60/83/38	31/85/43	
					rs8044769	55/71/39	63/90/21	
					rs17219084	57/82/43	52/120/36	
					rs8061518	85/73/21	107/82/17	
					rs9302652	104/67/11	103/86/20	
					rs9934773	103/66/11	88/66/17	
					rs17818866	99/70/15	84/71/16	
					rs17818902	101/66/14	102/88/17	
					rs8061228	108/70/2	134/73/2	
					rs10521303	54/91/37	75/101/29	
					rs12932428	55/85/41	53/105/50	
					rs9924877	98/68/14	91/89/29	
					rs7203181	93/76/13	85/94/30	
					rs1344502	57/85/37	55/107/45	
					rs9921255	124/56/2	111/83/10	
					rs6499662	100/68/14	106/84/18	
					rs7205987	129/48/6	144/53/7	
					rs1125338	41/98/39	53/102/51	
					rs2192872	86/76/17	100/84/25	
					rs708258	45/87/47	63/89/53	
					rs12599672	138/41/4	149/52/4	
					rs11076017	54/84/40	62/93/53	
					rs2665271	47/82/52	58/98/50	
X.‐R. Meng [43]	Obesity/Overweight	Children	1423/607	China	rs62048402	1054/330/31	469/128/7	MassARRAY
					rs9939609	1053/336/34	474/126/7	
J. Olza [44]	Obesity	Children	292/242	Spain	rs8061518	159/107/22	95/108/32	GoldenGate
					rs17818902	162/107/22	145/84/13	
					rs7190053	190/87/13	165/67/7	
					rs2111114	175/107/10	151/81/9	
					rs8044353	259/31/1	219/23/0	
					rs10521303	91/151/48	64/133/42	
					rs1558756	86/152/54	69/132/41	
					rs16952623	215/72/3	181/58/2	
					rs16952624	289/1/0	242/0/0	
					rs2111113	247/45/0	208/30/2	
					rs10852525	229/59/1	194/41/5	
					rs7194336	98/144/50	63/131/48	
					rs7203181	142/111/39	101/109/32	
					rs6499656	218/67/7	189/48/5	
					rs7191513	98/145/49	82/110/50	
					rs7194907	81/153/57	65/105/67	
					rs8056299	104/140/47	77/116/49	
					rs17225435	225/61/6	194/46/2	
					rs8049235	100/156/34	91/108/43	
					rs7199716	101/146/44	91/116/35	
					rs13334214	178/102/6	155/71/13	
					rs7194243	180/93/19	147/87/8	
					rs1136002	135/130/27	109/111/22	
					rs4784351	154/113/20	122/96/16	
					rs2540781	225/54/13	190/47/5	
					rs8049933	231/52/7	195/41/4	
					rs1558687	169/107/15	134/92/14	
					rs2075202	277/14/1	231/11/0	
					rs7200579	257/33/2	216/25/1	
					rs697771	102/137/53	72/127/43	
					rs12596638	221/66/4	176/61/5	
					rs1008400	81/148/61	67/132/43	
					rs12932373	214/76/2	175/61/3	
					rs2689248	79/153/59	64/113/63	
					rs17833492	140/127/24	106/112/23	
S. K. Vasan [45]	Obesity/Overweight	Adolescents	181/1036	India	rs9939609	93/58/30	487/403/146	Real‐time
Y. Yang [46]	Obesity	Children/Adolescents	170/200	China	rs9939609	117/51/2	163/35/2	Real‐time
					rs9935401	120/48/2	163/35/2	
B. Riffo [47]	Obesity	Children	238/136	Chile	rs9939609	111/67/60	73/47/16	Real‐time
A. Shahid [48]	Obesity	Children	78/45	Pakistan	rs9939609	50/26/2	30/15/0	PCR‐RFLP
L. Wu [49]	Obesity	Children	1229/1619	China	rs6499640	813/350/45	1130/413/45	Real‐time
B. Xi [50]	Obesity/Overweight	Children/Adolescents	1229/2274	China	rs9939609	915/288/26	1803/436/35	Real‐time
P. Zavattari [51]	Obesity	Children/Adolescents	912/543	Italy	rs9939609	197/430/285	183/254/106	Real‐time
L. Wang [52]	Obesity	Children	150/389	China	rs1421085	107/37/6	315/67/7	PCR‐RFLP
					rs17817449	104/40/6	289/93/7	
Y. Xu [53]	Obesity	Children	499/489	China	rs4784323	291/182/26	277/179/33	SNPScan
					rs7206790	345/139/15	364/121/4	
					rs1477196	292/181/26	292/174/23	
					rs3751813	229/216/54	248/200/40	
					rs9939811	137/247/115	118/254/117	
					rs9924072	295/178/26	284/178/27	
					rs12919344	280/187/31	252/197/39	
					rs11644943	363/133/3	353/124/12	
					rs12446047	278/196/25	264/195/30	
					rs9932411	283/189/27	280/187/22	
					rs7206456	22,148/230/48	216/215/58	
					rs9302654	387,106/6	380/104/5	
					rs16952730	258/202/39	248/204/37	
					rs6499661	433/64/2	443/45/1	
					rs7199716	206/216/77	179/221/89	
					rs13335453	299/170/30	305/165/19	
					rs1971037	146/240/113	128/233/127	
					rs7184897	158/234/107	135/235/118	
					rs12596638	215/221/63	210/219/60	
					rs3928987	185/238/76	176/242/71	
					rs708255	176/241/82	171/237/81	
					rs741300	146/250/103	132/259/98	
					rs17236863	458/38/3	442/45/2	
P. Todendi [54]	Overweight	Children/Adolescents	530/1441	Brazil	rs9939609	186/262/82	580/664/197	TaqMan
	Obesity		386/1441			131/177/78	580/664/197	
J. Wu [55]	Overweight	Adolescents	178/223	China	rs9939609	131/42/3	179/40/1	MassARRAY
					rs1558902	131/42/3	178/41/1	
					rs8050136	131/43/3	179/40/1	
					rs3751812	131/43/3	178/40/1	
					rs6499640	126/44/6	159/54/8	
M. Yang [56]	Obesity	Adolescents	1348/2576	China	rs9939609	951/356/41	2031/519/26	Real‐time
H. Abd Ali [57]	Obesity	Children/Adolescents	300/200	Iraq	rs9939609	60/149/91	31/82/87	AS‐PCR
E. Bryl [58]	Overweight	Children	70/317	Poland	rs9939609	25/32/13	104/146/67	Real‐time
L. Dušátková [59]	Obesity/Overweight	Adolescents	666/766	Czech	rs9939609	152/338/176	240/387/139	TaqMan
M. Zhang [60]	Obesity	Children	757/2746	China	rs9939609	567/170/20	2150/555/41	AS‐PCR
N. Kalantari [61]	Obesity/Overweight	Adolescents	98/130	Iran	rs9930506	29/39/30	49/73/8	Sequencing

Abbreviations: HET, heterozygous; M, homozygous mutant; W, homozygous wild‐type.

### 3.8 Publication Bias

A funnel plot was utilised to examine the publication bias of the included articles. These curves demonstrate the correlation between polymorphisms and the risk of obesity in allelic, dominant, recessive and co‐dominant genetic analysis models. The symmetrical shape of the funnel plots indicated that there is no publication bias in our study of FTO polymorphisms. Also, and based on our analysis (only for rs9939609 in obesity subgroup), the Egger test was insignificant for all models. The output of our analysis in this respect is presented in Figure S6.

### 3.9 Sensitivity Analysis

In most included studies, the genotypes are compatible with HWE which was interesting to be statistically significant at *p* ≤ 0.05. To exclude the effect of incompatible HWE studies, we conducted a sensitivity analysis. Accordingly, after removing the studies with incompatible HWE, the pooled estimates of all models were increased for rs9939609 in all genetic models (Figure S7–S11). Moreover, after careful inspection of the data set, it was realised that, if we applied the same strategy to other SNPs, the possibility of conducting meta‐analysis will be violated. The rs1421085 was an exception, since in all the studies, the HWE was confirmed for this particular SNP.

## 4 Discussion

In our meta‐analysis study, only rs9939609 is related to both obesity and overweight children and adolescents, and we observed significance association in the recessive model. rs1421085 shown similar result just in obese group. The overall pooled OR in homozygote model of obesity risk was 1.34 and also pooled OR of overweight/obese risk in recessive model was 1.61. In Liu, Mou, and Cai [9] studies, meta‐analysis pooled OR in additive model reported 1.35 (95% CI: 1.25–1.47) in obese children and adolescents. Moreover, a positive correlation between FTO SNPs and obesity was observed in children, based on subgroup analysis results in a meta‐analysis of Asian obesity risk [65].

In this meta‐analysis, we confirmed that a variant in the FTO gene is significantly associated with an increased risk of overweight or obesity in children and adolescents. Publication bias analysis confirmed that there is a positive relationship among different ethnicities; however, heterogeneity between studies is common in genetic association meta‐analyses [66]. Subgroup analysis was performed to evaluate heterogeneity based on ethnicity and different variants. Different studies shown that FTO polymorphism have been related to obesity and overweight.

Unlike our study, a meta‐analysis in Caucasian individuals under 18 years old showed a more significant association of rs9939609 in the co‐dominant models (AA vs. TT) with OR = 1.91 (95% CI: 1.47–2.48). This study comprised of 5000 obese children/adolescents, and 9853 controls were investigated from 12 eligible studies [67], while there was no association found between rs9939609 and metabolic syndrome in children/adolescents [68].

In this present study, FTO SNPs rs9939609, rs1421085, rs1861868, rs1477196 and rs17817449 are pooled in obese children and adolescents. However, according to a recent study in the Chinese population under the age of 18, FTO SNPs such as rs9939609, rs8050136, rs6499640 and rs1558902 have been found to be strongly linked to an increased risk of obesity [69]. But our study found no strong association between overweight and FTO SNP rs6499640 in children and adolescents (Figure 4). In Chinese girls aged 6–18, the association between the SNP rs6499640 and lipid profiles was observed (*p* < 0.001); this association was not seen in boys [70].

The FTO SNP rs17817449 showed a positive association with BMI, waist circumference, fasting insulin and glucose. Additionally, the GT and GG genotypes show a strong association with obesity (OR: 1.75, 95% CI: 1.02–3.02). The G allele (minor allele) can be used to predict the risk of type 2 diabetes in obese Egyptian children/adolescents [71]. However, no correlation was observed between rs17817449 and rs9939609 and obesity among obese Egyptian children/adolescents [38]. Furthermore, we found no association between rs17817449 and obesity in various genetic models. Odds ratio in the recessive model was 0.96, 95% CI: 0.21; 4.38.

The exact mechanism by which FTO variants increase the risk of obesity is still unclear. Some studies have shown that the impact of FTO variants on BMI may be attributed to their influence on total calorie intake, frequency of food consumption or preference for certain types of nutrients, such as fat or protein [23, 72, 73]. The study found a significant link between rs9939609 and rs1421085 and the total energy intake in children and adolescents [74]. An analysis of 16,094 children and adolescents from 14 eligible studies showed that rs9939609 was associated with total energy and macronutrient intake as well as BMI [75]. Intronic FTO SNPs can impact IRX3 (Iroquois homeobox 3), a crucial protein for neural system development, therefore potentially affecting hunger signals and satiety [76].

### 4.1 Limitation and Strengths

To the best of our knowledge, this meta‐analysis is the first to comprehensively evaluate the association of FTO gene polymorphisms with childhood obesity. Previous studies mainly focused on the effect of FTO rs9939609 on childhood obesity. However, in this study, we aimed to provide a comprehensive overview of the other FTO gene polymorphisms alongside FTO rs9939609 itself. Decentralising the focus from one polymorphism to the others is important as it allows for consideration of their association and opens up new research avenues. This is critical because our study revealed that many other polymorphisms showed a stronger association with obesity and overweight than FTO rs9939609 in the same studies.

Despite these important findings, there are still some limitations in this study that should be acknowledged. Firstly, only limited ethnicities were included which influenced the level of association observed. Therefore, conducting GWAS studies in different ethnicities and considering multiple polymorphisms can provide a more precise and comprehensive understanding of the association between FTO gene polymorphisms and childhood obesity.

Furthermore, obesity is a complex disorder and focusing only on genetic factors may provide a biased view of the disease's pathophysiology. Therefore, it is crucial to include other factors such as behavioural and environmental parameters in the analysis. Unfortunately, due to lack of data, our study was unable to measure the effects of other parameters such as environment and diet factors alongside their interactions with genetic parameters.

## 5 Conclusions

According to our meta‐analysis and systematic study, we have found that the FTO rs9939609 and rs1421085 are associated with an increased risk of obesity among children and adolescents. However, further research on larger populations and gene–environment interaction studies is necessary to confirm these findings and gain a better understanding of the FTO gene's function in relation to overweight/obesity in children and adolescents.

## Author Contributions

S.C. conceived the study, designed and undertook the study. S.C., M.E. and S.T. were involved in paper screening, data extraction and interpreted the results, as well as drafted and edited the first draft. Bibliographic search was performed by S.C. and A.M. Data analysis was carried out by S.C. and A.M. S.C. wrote the first draft of the manuscript. All authors read and approved the final version of the paper.

## Disclosure

The corresponding author of this work, Dr. Sara Cheraghi, received financial support from research deputy of the Iran University of Medical Sciences. Other authors have no competing interests to report.

## Ethics Statement

The authors have nothing to report.

## Supporting information


Figures S1–S11.

Table S1.

## Data Availability

All the data used in this study can be obtained from forest plots and tables. Nonetheless, the data set of this work is available from corresponding author upon request.
