# Supplementary material for: Genetic Variants in the Fat Mass and Obesity‐Associated Gene and Risk of Obesity/Overweight in Children and Adolescents: A Systematic Review and Meta‐Analysis
Source: Endocrinol Diabetes Metab. 2024 Jul 7;7(4):e00510. doi: 10.1002/edm2.510 (PMC11227992; doi:10.1002/edm2.510)
Supplement: Supplementary file 1 — Figures S1–S11. [file EDM2-7-e00510-s001.docx]

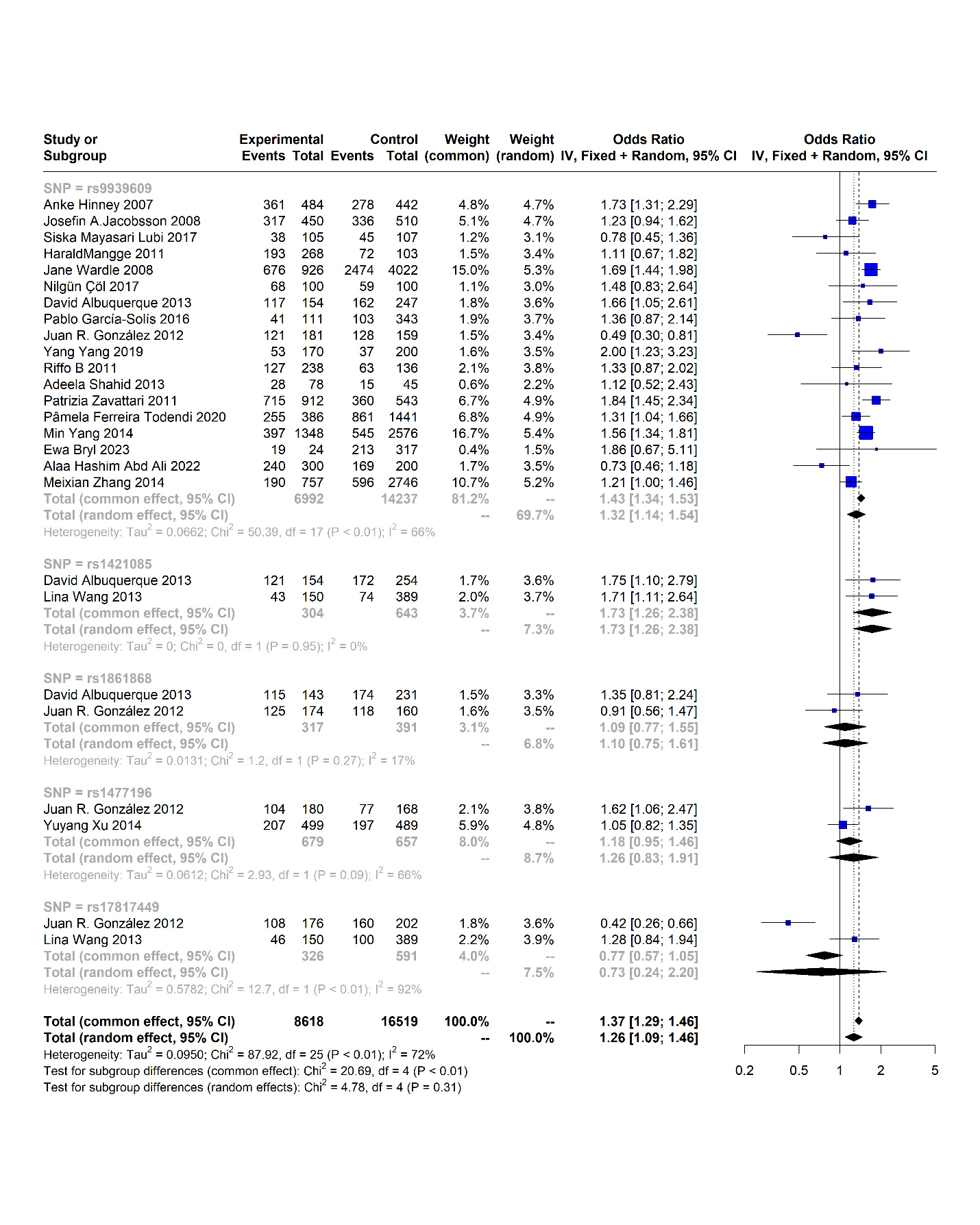


Supplementary Figure 1: Forest plot in obese children and adolescents in model dominant


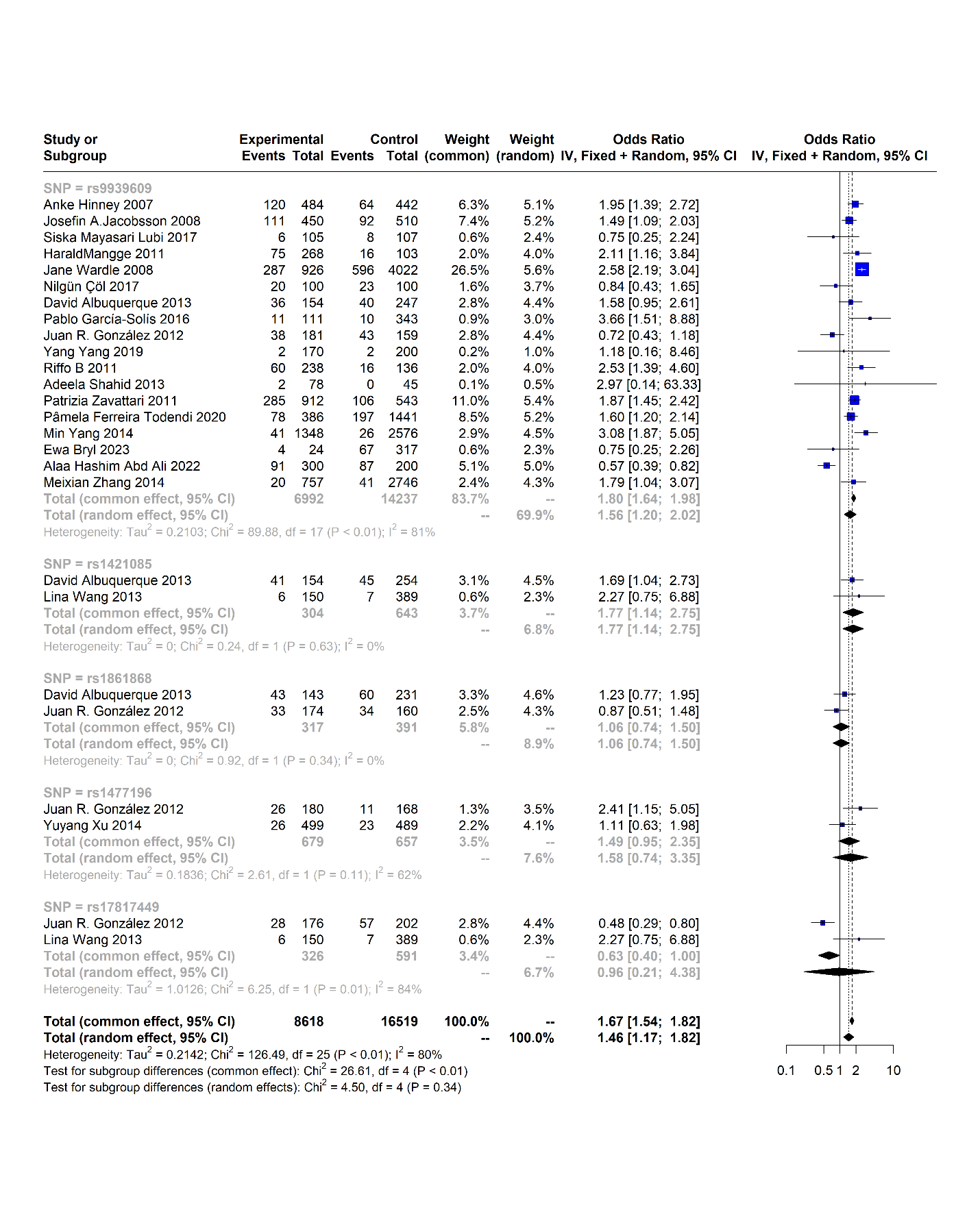


Supplementary Figure *2*. Forest plot in obese children and adolescents in model recessive


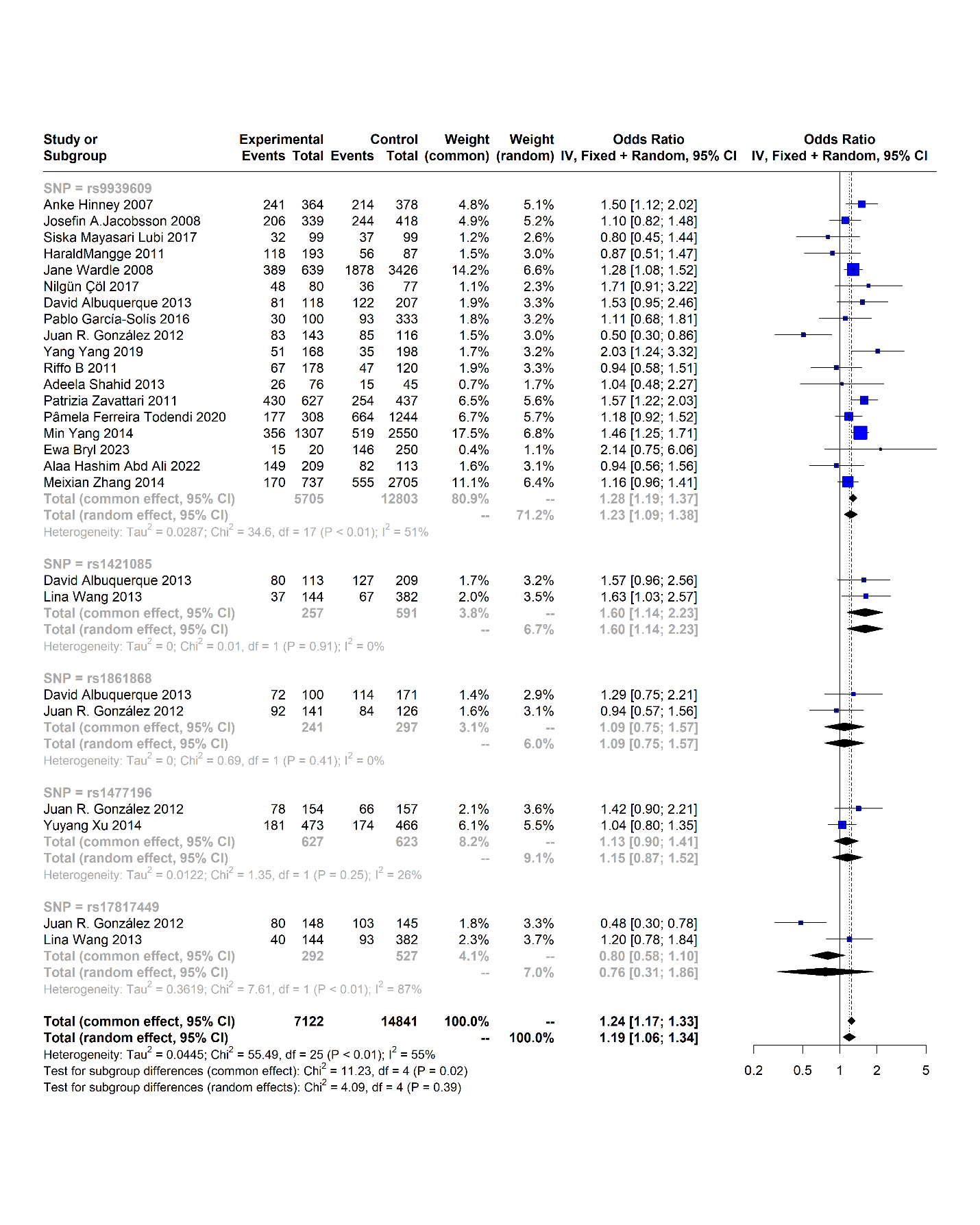


Supplementary Figure 3. Forest plot in obese children and adolescents in Aa vs aa model


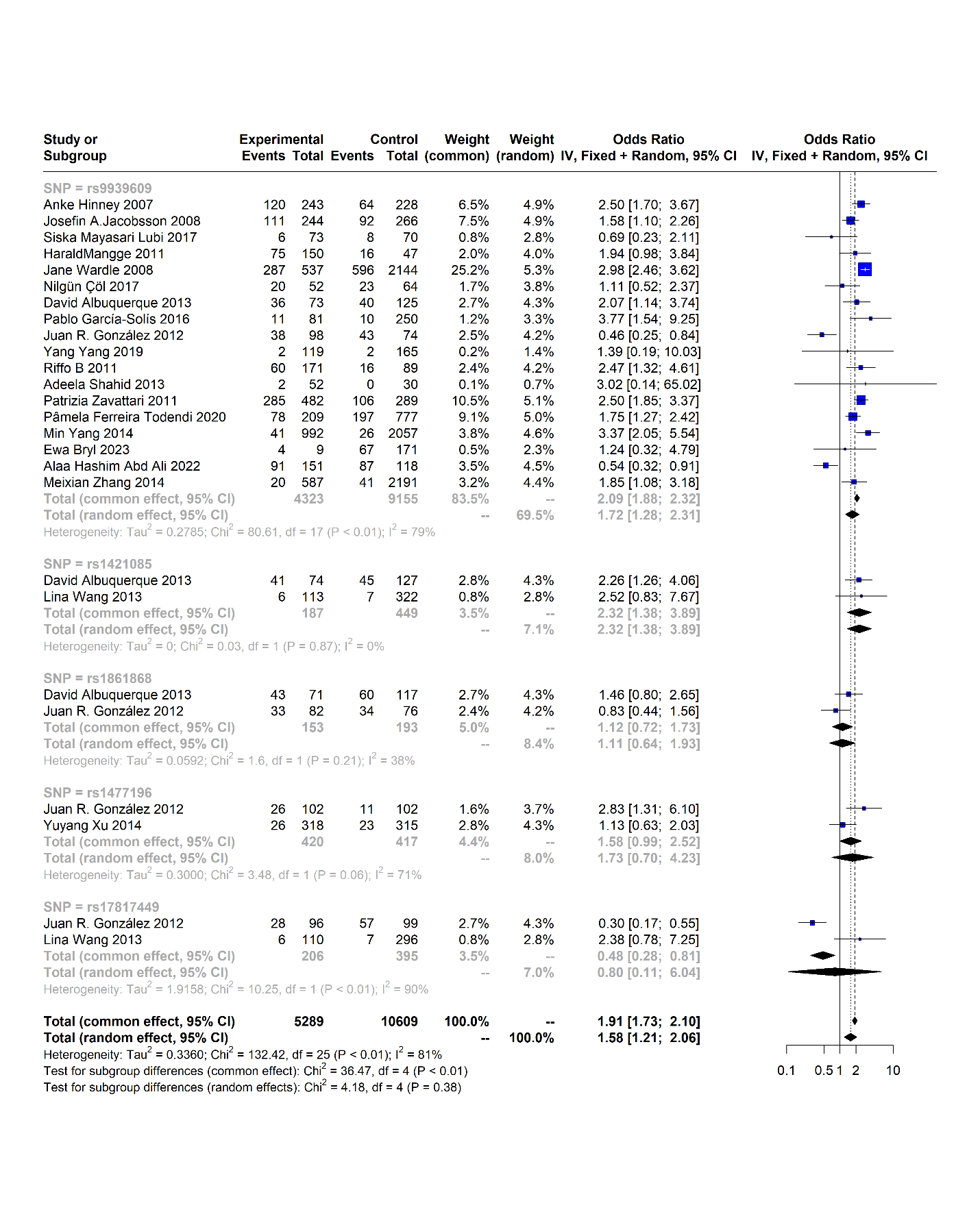


Supplementary Figure 4. Forest plot in obese children and adolescents in AA vs aa model


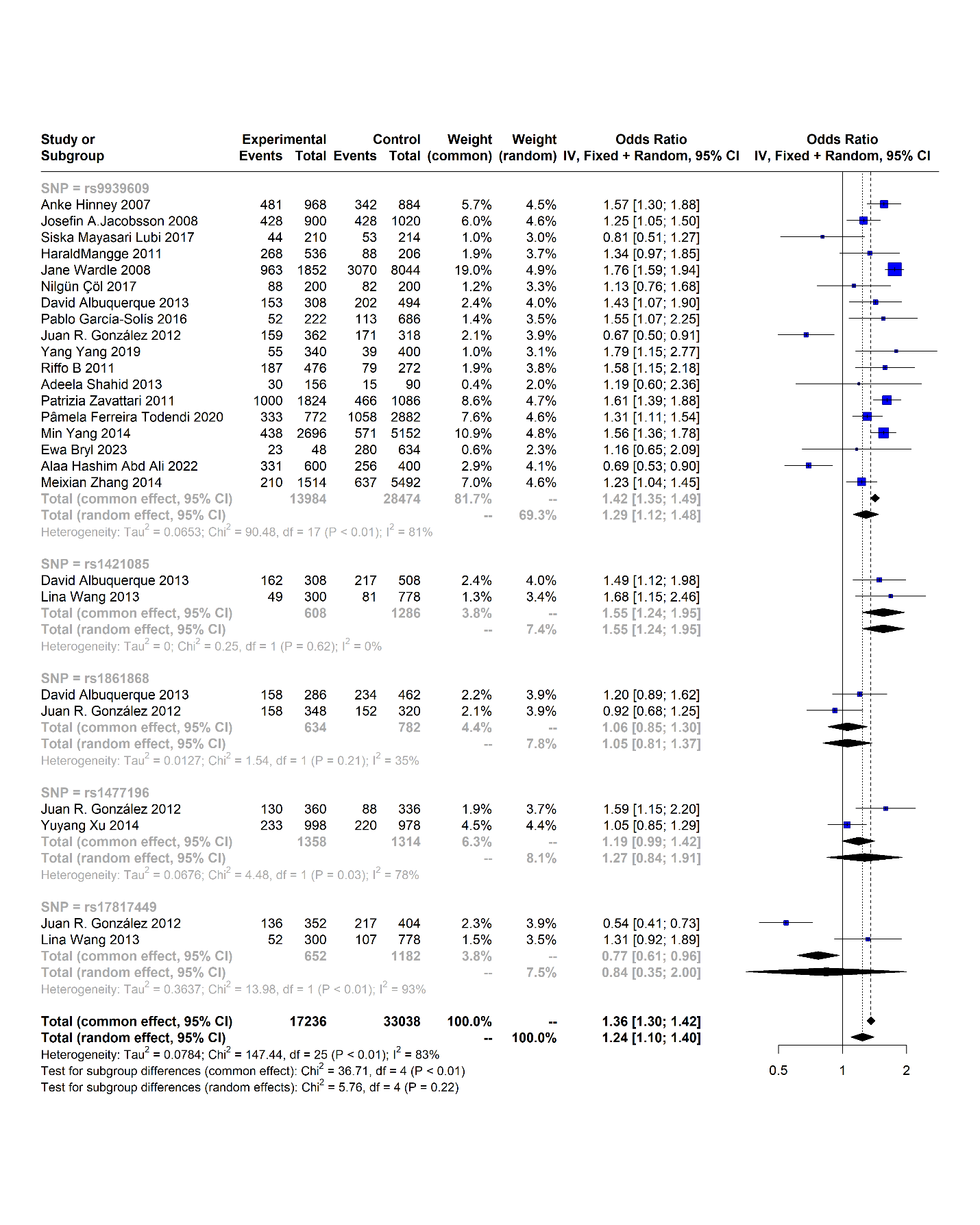


Supplementary Figure 5. Forest plot in obese children and adolescents in allelic model


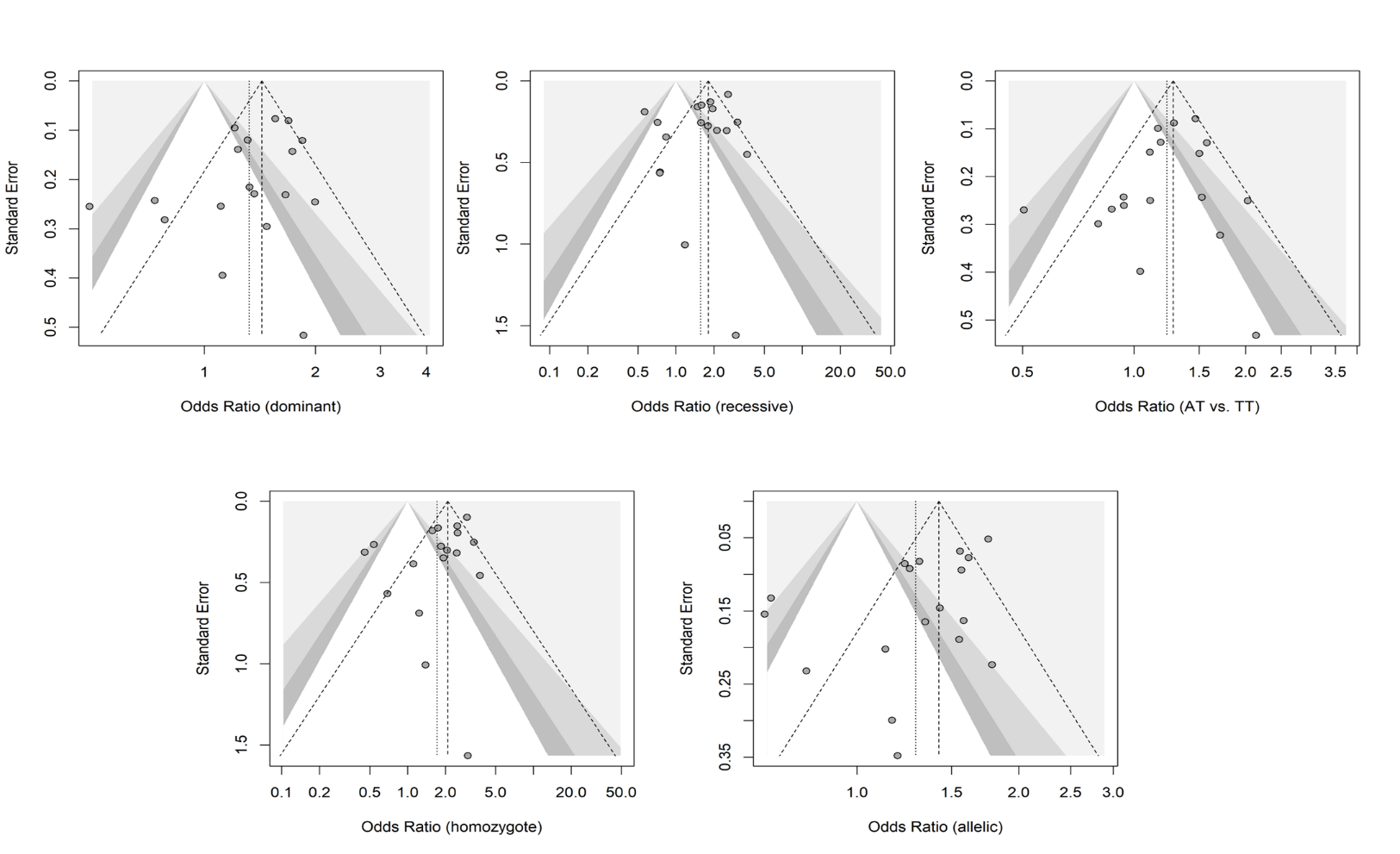


Supplementary Figure 6. Funnel plots to investigate publication bias in dominant (P_egger_=0.1015), recessive (P_egger_=0.1681), AA vs.TT (P_egger_=0.2352), homozygote (P_egger_=0.0818), allelic (P_egger_=0.1120). All the funnels belong to the rs9939609 in the obesity subgroup.


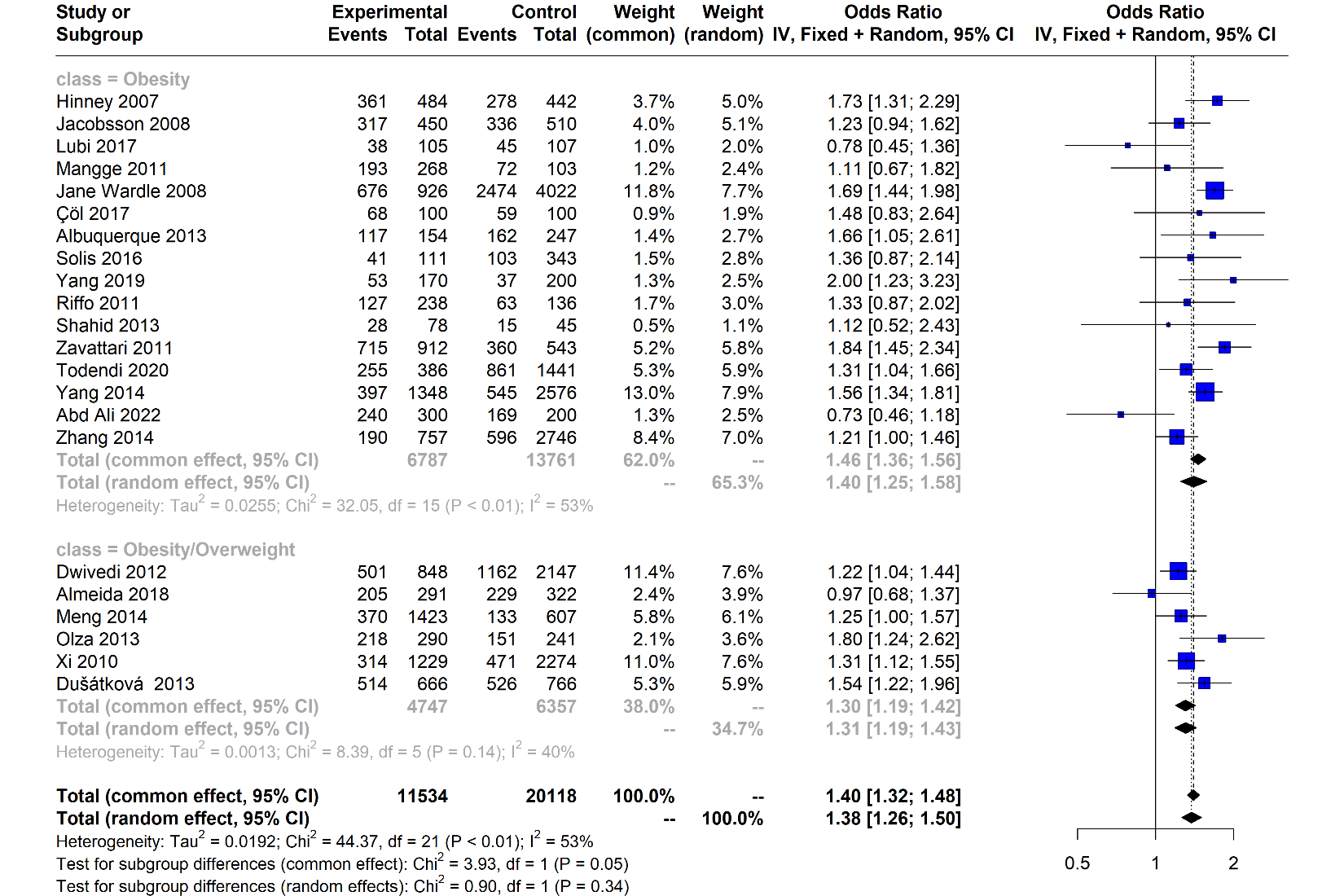


Supplementary Figure 7. Sensitivity analysis for studies in which HWE was confirmed in model dominant


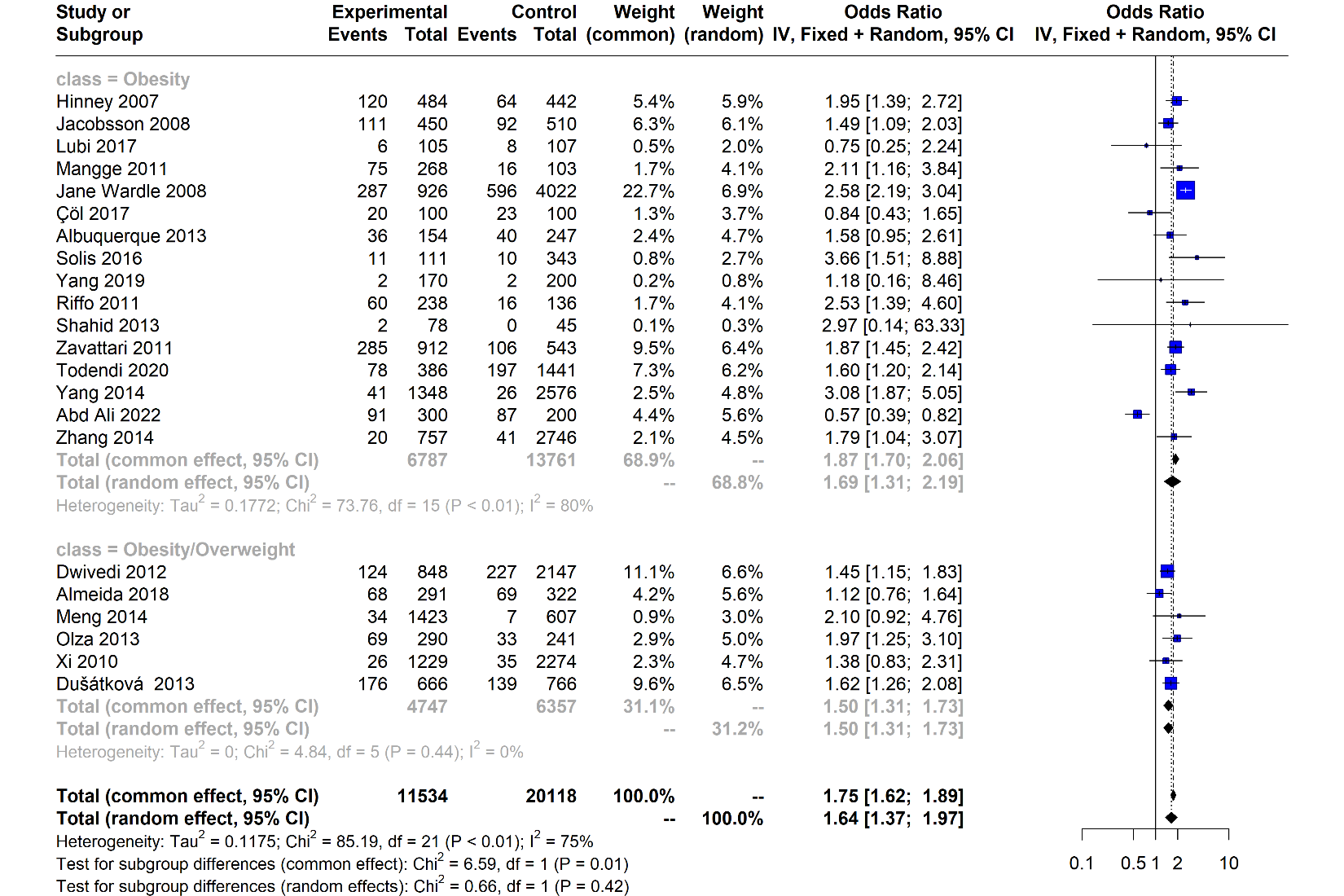


Supplementary Figure 8. Sensitivity analysis for studies in which HWE was confirmed in model recessive


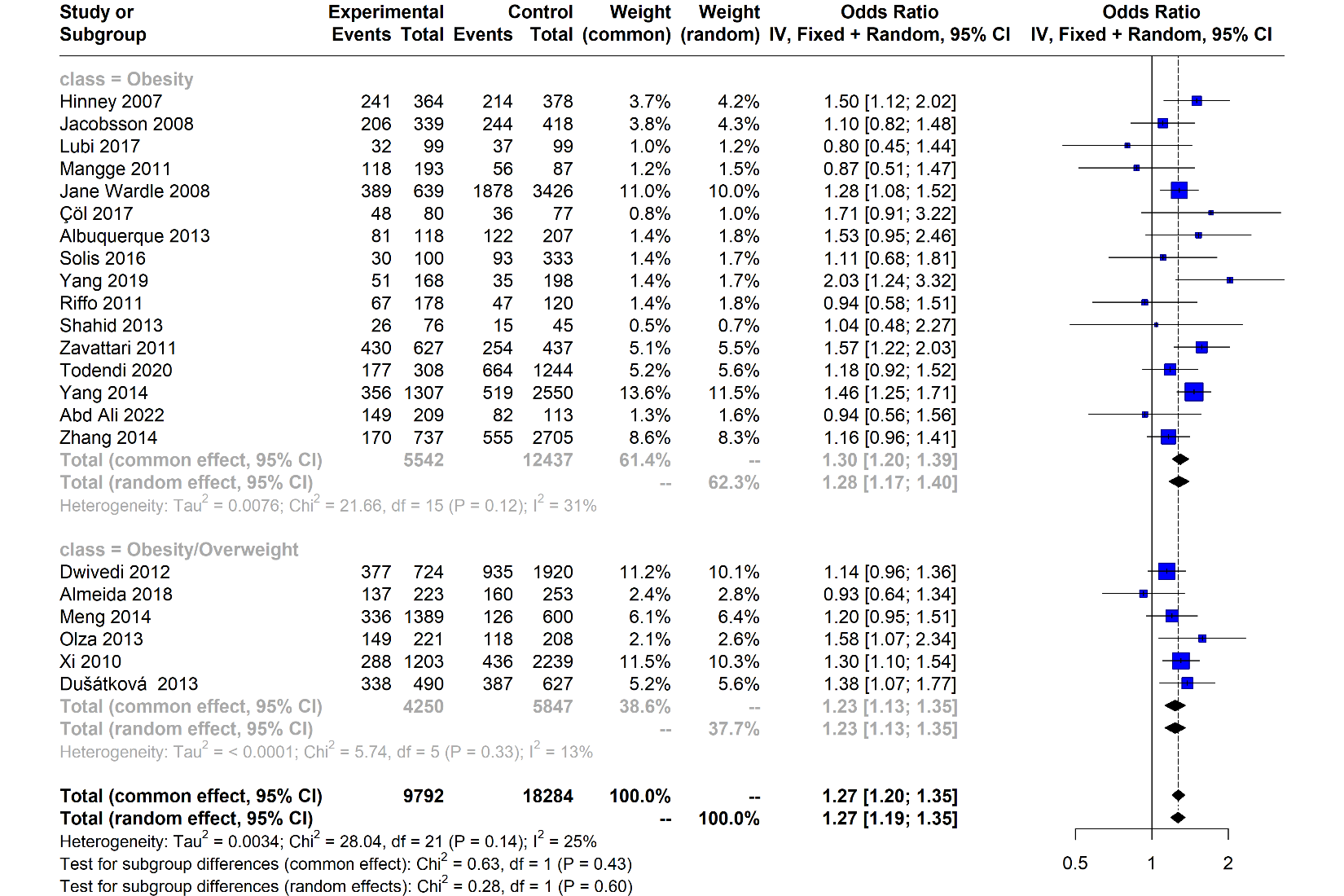


Supplementary Figure 9. Sensitivity analysis for studies in which HWE was confirmed in model AT vs. TT


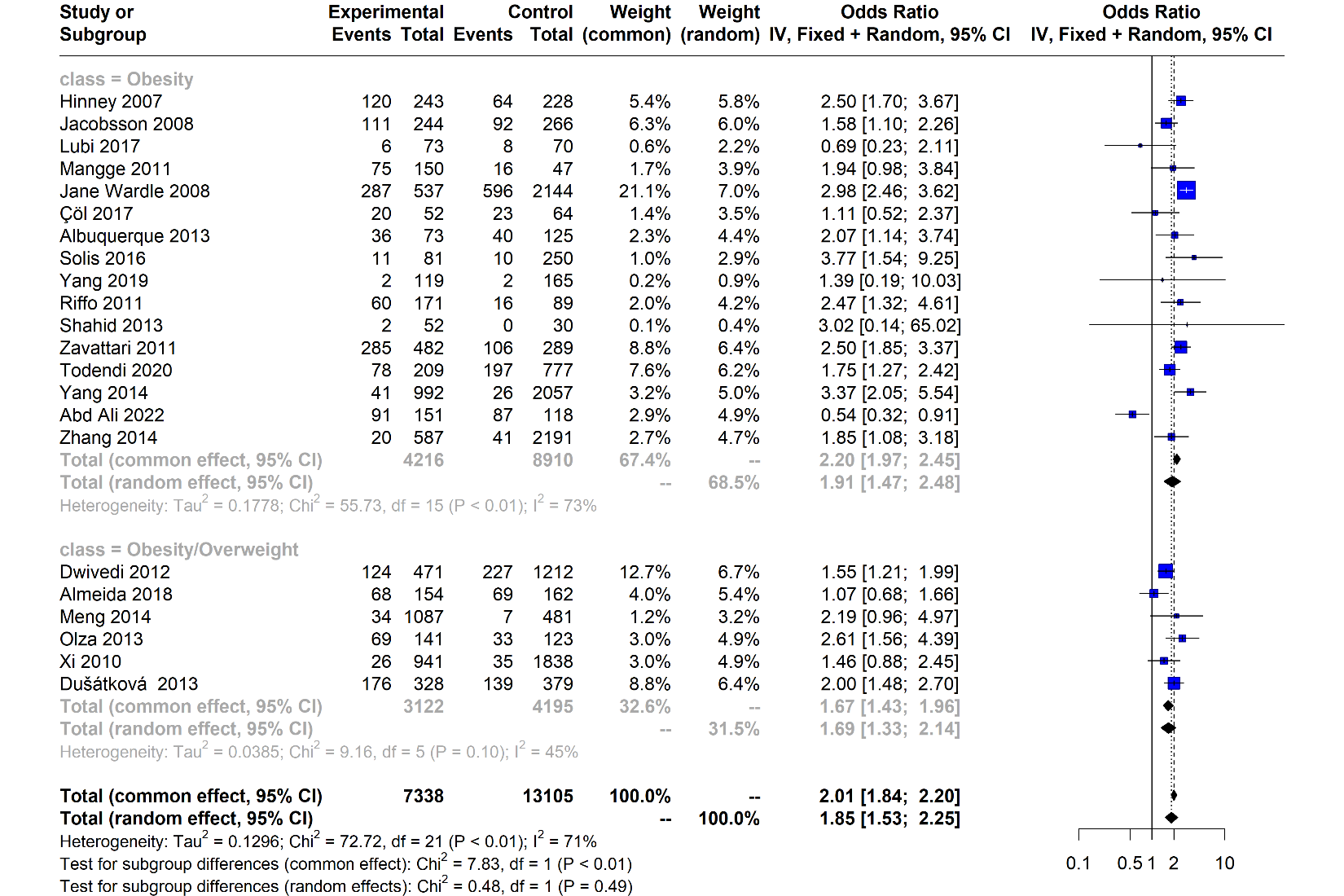


Supplementary Figure 10. Sensitivity analysis for studies in which HWE was confirmed in model homozygote


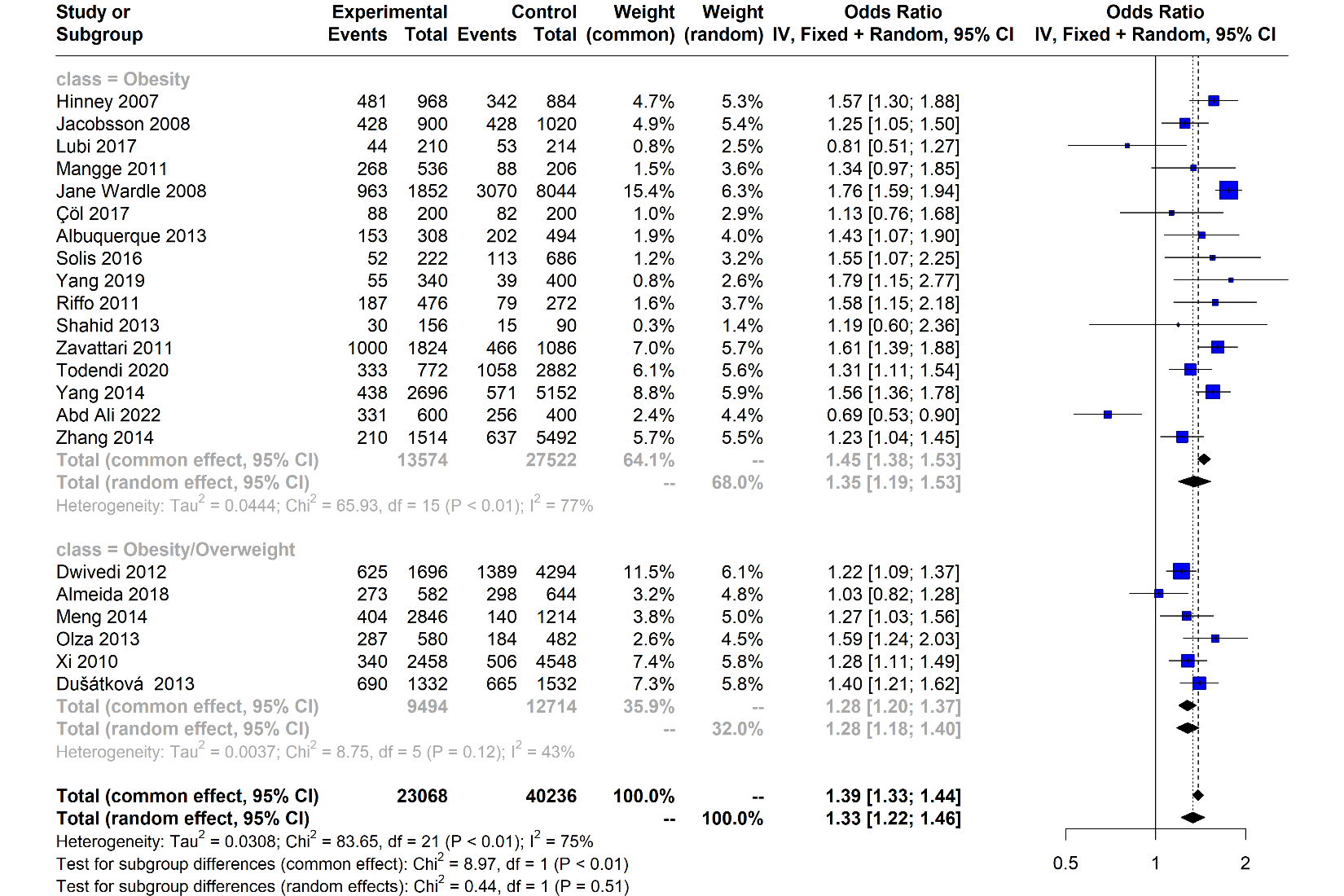


Supplementary Figure 11. Sensitivity analysis for studies in which HWE was confirmed in allelic model
