# Supplementary material for: Genetic Variants in the Fat Mass and Obesity‐Associated Gene and Risk of Obesity/Overweight in Children and Adolescents: A Systematic Review and Meta‐Analysis
Source: Endocrinol Diabetes Metab. 2024 Jul 7;7(4):e00510. doi: 10.1002/edm2.510 (PMC11227992; doi:10.1002/edm2.510)
Supplement: Supplementary file 2 — Table S1. [file EDM2-7-e00510-s002.docx]

Supplementary Table 1. Candidate SNPs were considered overweight/obese only in one reports

| Author/Year | Country | Study design | Sample size (case/control) | SNP | Risk allele | Method | OR (95%CI)* |
| --- | --- | --- | --- | --- | --- | --- | --- |
| Obesity |  |  |  |  |  |  |  |
| Hinney A (2007) | Germany | Case-Control | 487/442 | rs1121980 | T | MALDI-TOF MS | **1.66 (1.37;2.02)^a^** |
| Hinney A (2007) | Germany | Case-Control | 487/442 | rs9939973 | A | MALDI-TOF MS | **1.64 (1.36;1.99)^a^** |
| Hinney A (2007) | Germany | Case-Control | 487/442 | rs7193144 | C | MALDI-TOF MS | **1.59 (1.32;1.93)^b^** |
| Hinney A (2007) | Germany | Case-Control | 487/442 | rs9940128 | A | MALDI-TOF MS | **1.61 (1.32;1.95)^a^** |
| Gonzalez JR (2012) | Spain | Case-Control | 202/184 | rs2241179 | C | TaqMan | 0.70 (0.35;1.39)^c^ |
| Gonzalez JR (2012) | Spain | Case-Control | 202/184 | Rs1477199 | G | TaqMan | 0.35 (0.09;1.39)^c^ |
| Gonzalez JR (2012) | Spain | Case-Control | 202/184 | rs8044769 | T | TaqMan | 1.20 (0.74;1.95)^c^ |
| Gonzalez JR (2012) | Spain | Case-Control | 202/184 | rs17219084 | G | TaqMan | 1.57 (0.52;1.71)^c^ |
| Gonzalez JR (2012) | Spain | Case-Control | 202/184 | rs9302652 | C | TaqMan | 1.85 (0.83; 4.11)^c^ |
| Gonzalez JR (2012) | Spain | Case-Control | 202/184 | rs9934773 | T | TaqMan | 1.86 (0.82; 4.24)^c^ |
| Gonzalez JR (2012) | Spain | Case-Control | 202/184 | rs17818866 | G | TaqMan | 1.26 (0.58; 2.74)^c^ |
| Gonzalez JR (2012) | Spain | Case-Control | 202/184 | rs8061228 | C | TaqMan | 1.00 (0.14; 7.43)^c^ |
| Gonzalez JR (2012) | Spain | Case-Control | 202/184 | rs12932428 | T | TaqMan | 1.33 (0.82; 2.16)^c^ |
| Gonzalez JR (2012) | Spain | Case-Control | 202/184 | rs9924877 | A | TaqMan | 2.00 (0.98; 4.07)^c^ |
| Gonzalez JR (2012) | Spain | Case-Control | 202/184 | rs1344502 | C | TaqMan | 1.38 (0.86; 2.23)^c^ |
| Gonzalez JR (2012) | Spain | Case-Control | 202/184 | rs9921255 | C | TaqMan | **5.78 (1.22; 27.5)^c^** |
| Gonzalez JR (2012) | Spain | Case-Control | 202/184 | rs6499662 | G | TaqMan | 1.17 (0.54; 2.51)^c^ |
| Gonzalez JR (2012) | Spain | Case-Control | 202/184 | rs7205987 | C | TaqMan | 1.05 (0.34; 3.26)^c^ |
| Gonzalez JR (2012) | Spain | Case-Control | 202/184 | rs1125338 | C | TaqMan | 1.11 (0.61; 2.02)^c^ |
| Gonzalez JR (2012) | Spain | Case-Control | 202/184 | rs2192872 | C | TaqMan | 1.23 (0.62; 2.46)^c^ |
| Gonzalez JR (2012) | Spain | Case-Control | 202/184 | rs708258 | G | TaqMan | 0.76 (0.43; 1.33)^c^ |
| Gonzalez JR (2012) | Spain | Case-Control | 202/184 | rs12599672 | A | TaqMan | 1.29 (0.80; 2.09)^c^ |
| Gonzalez JR (2012) | Spain | Case-Control | 202/184 | rs11076017 | C | TaqMan | 1.22 (0.69; 2.13)^c^ |
| Gonzalez JR (2012) | Spain | Case-Control | 202/184 | rs2665271 | G | TaqMan | 0.68 (0.39; 1.20)^c^ |
| Gonzalez JR (2012) | Spain | Case-Control | 202/184 | rs7203521 | G | TaqMan | 0.70 (0.35;1.40)^c^ |
| Wu L (2010) | China | Cross sectional | 1229/1619 | rs6499640 | A | TaqMan | **1.16 (1.10; 1.21)^b^** |
| Xu Y (2014) | China | Cross sectional | 499/489 | rs4784323 | A | SNPScan | NA |
| Xu Y (2014) | China | Cross sectional | 499/489 | rs72066790 | G | SNPScan | **3.97 (1.30; 12.10)^c^** |
| Xu Y (2014) | China | Cross sectional | 499/489 | rs3751813 | T | SNPScan | NA |
| Xu Y (2014) | China | Cross sectional | 499/489 | rs9939811 | T | SNPScan | NA |
| Xu Y (2014) | China | Cross sectional | 499/489 | rs9924072 | G | SNPScan | NA |
| Xu Y (2014) | China | Cross sectional | 499/489 | rs12919344 | A | SNPScan | NA |
| Xu Y (2014) | China | Cross sectional | 499/489 | rs11644943 | A | SNPScan | **0.16 (0.04; 0.73)^c^** |
| Xu Y (2014) | China | Cross sectional | 499/489 | rs12446047 | C | SNPScan | NA |
| Xu Y (2014) | China | Cross sectional | 499/489 | rs9932411 | C | SNPScan | NA |
| Xu Y (2014) | China | Cross sectional | 499/489 | rs7206456 | A | SNPScan | NA |
| Xu Y (2014) | China | Cross sectional | 499/489 | rs9302654 | T | SNPScan | NA |
| Xu Y (2014) | China | Cross sectional | 499/489 | rs16952730 | G | SNPScan | NA |
| Xu Y (2014) | China | Cross sectional | 499/489 | rs6499661 | T | SNPScan | NA |
| Xu Y (2014) | China | Cross sectional | 499/489 | rs13335453 | G | SNPScan | NA |
| Xu Y (2014) | China | Cross sectional | 499/489 | rs1971037 | T | SNPScan | NA |
| Xu Y (2014) | China | Cross sectional | 499/489 | rs7184897 | A | SNPScan | NA |
| Xu Y (2014) | China | Cross sectional | 499/489 | rs3928987 | A | SNPScan | NA |
| Xu Y (2014) | China | Cross sectional | 499/489 | rs708255 | A | SNPScan | NA |
| Xu Y (2014) | China | Cross sectional | 499/489 | rs741300 | G | SNPScan | NA |
| Xu Y (2014) | China | Cross sectional | 499/489 | rs17236863 | T | SNPScan | NA |
| Xu Y (2014) | China | Cross sectional | 499/489 | rs12596638 | A | SNPScan | NA |
| Xu Y (2014) | China | Cross sectional | 499/489 | rs7199716 | T | SNPScan | NA |
| Overweight |  |  |  |  |  |  |  |
| Wu J (2014) | China | Case-Control | 178/223 | rs1558902 | A | MassARRAY | 1.46 (0.94; 2.26)^b^ |
| Wu J (2014) | China | Case-Control | 178/225 | rs3751812 | T | MassARRAY | 0.66 (0.43; 1.02)^b^ |
| Obesity/Overweight |  |  |  |  |  |  |  |
| Olza J (2013) | Spain | Case-Control | 292/242 | rs12445162 | A | GoldenGate Assay | 0.63 (0.35; 1.11)^c^ |
| Olza J (2013) | Spain | Case-Control | 292/242 | rs11075986 | G | GoldenGate Assay | 1.07 (0.80; 1.44)^c^ |
| Olza J (2013) | Spain | Case-Control | 292/242 | rs11643744 | G | GoldenGate Assay | 0.86 (0.61; 1.22)^c^ |
| Olza J (2013) | Spain | Case-Control | 292/242 | rs9928094 | G | GoldenGate Assay | **1.66 (1.22; 2.24)^c^** |
| Olza J (2013) | Spain | Case-Control | 292/242 | rs9930333 | G | GoldenGate Assay | **1.64 (1.21; 2.22)^c^** |
| Olza J (2013) | Spain | Case-Control | 292/242 | rs7205986 | G | GoldenGate Assay | 0.78 (0.46; 1.34)^c^ |
| Olza J (2013) | Spain | Case-Control | 292/242 | rs3826169 | C | GoldenGate Assay | 1.32 (0.92; 1.89)^c^ |
| Olza J (2013) | Spain | Case-Control | 292/242 | rs7190053 | T | GoldenGate Assay | 1.21 (0.82; 1.78)^c^ |
| Olza J (2013) | Spain | Case-Control | 292/242 | rs2111114 | G | GoldenGate Assay | 1.19 (0.82; 1.74)^c^ |
| Olza J (2013) | Spain | Case-Control | 292/242 | rs8044353 | A | GoldenGate Assay | 1.21 (0.61; 2.39)^c^ |
| Olza J (2013) | Spain | Case-Control | 292/242 | rs1558756 | T | GoldenGate Assay | 1.14 (0.83; 1.57)^c^ |
| Olza J (2013) | Spain | Case-Control | 292/242 | rs16952623 | C | GoldenGate Assay | 0.98 (0.62; 1.56)^c^ |
| Olza J (2013) | Spain | Case-Control | 292/242 | rs16952624 | T | GoldenGate Assay | NA |
| Olza J (2013) | Spain | Case-Control | 292/242 | rs2111113 | C | GoldenGate Assay | 1.00 (0.56; 1.78)^c^ |
| Olza J (2013) | Spain | Case-Control | 292/242 | rs10852525 | A | GoldenGate Assay | 0.89 (0.55; 1.45)^c^ |
| Olza J (2013) | Spain | Case-Control | 292/242 | rs7194336 | T | GoldenGate Assay | 0.74 (0.55; 1.01)^c^ |
| Olza J (2013) | Spain | Case-Control | 292/242 | rs6499656 | C | GoldenGate Assay | 1.30 (0.84; 2.03)^c^ |
| Olza J (2013) | Spain | Case-Control | 292/242 | rs7191513 | A | GoldenGate Assay | 0.96 (0.72; 1.30)^c^ |
| Olza J (2013) | Spain | Case-Control | 292/242 | rs7194907 | C | GoldenGate Assay | 0.87 (0.64; 1.18)^c^ |
| Olza J (2013) | Spain | Case-Control | 292/242 | rs8056299 | G | GoldenGate Assay | 0.84 (0.62; 1.14)^c^ |
| Olza J (2013) | Spain | Case-Control | 292/242 | rs17225435 | G | GoldenGate Assay | 1.51 (0.93; 2.46)^c^ |
| Olza J (2013) | Spain | Case-Control | 292/242 | rs8049235 | A | GoldenGate Assay | 1.01 (0.74; 1.59)^c^ |
| Olza J (2013) | Spain | Case-Control | 292/242 | rs13334214 | T | GoldenGate Assay | 1.07 (0.74; 1.57)^c^ |
| Olza J (2013) | Spain | Case-Control | 292/242 | rs7194243 | T | GoldenGate Assay | 0.82 (0.57; 1.18)^c^ |
| Olza J (2013) | Spain | Case-Control | 292/242 | rs1136002 | C | GoldenGate Assay | 0.90 (0.66; 1.24)^c^ |
| Olza J (2013) | Spain | Case-Control | 292/242 | rs4784351 | G | GoldenGate Assay | 0.92 (0.65; 1.28)^c^ |
| Olza J (2013) | Spain | Case-Control | 292/242 | rs2540781 | A | GoldenGate Assay | 0.87 (0.57; 1.32)^c^ |
| Olza J (2013) | Spain | Case-Control | 292/242 | rs8049933 | T | GoldenGate Assay | 0.83 (0.52; 1.32)^c^ |
| Olza J (2013) | Spain | Case-Control | 292/242 | rs1558687 | T | GoldenGate Assay | 0.94 (0.67; 1.33)^c^ |
| Olza J (2013) | Spain | Case-Control | 292/242 | rs2075202 | G | GoldenGate Assay | 0.69 (0.25; 1.88)^c^ |
| Olza J (2013) | Spain | Case-Control | 292/242 | rs7200579 | G | GoldenGate Assay | 0.91 (0.50; 1.68)^c^ |
| Olza J (2013) | Spain | Case-Control | 292/242 | rs697771 | T | GoldenGate Assay | 1.00 (0.74; 1.36)^c^ |
| Olza J (2013) | Spain | Case-Control | 292/242 | rs1008400 | T | GoldenGate Assay | 0.97 (0.71; 1.32)^c^ |
| Olza J (2013) | Spain | Case-Control | 292/242 | rs12932373 | T | GoldenGate Assay | 0.77 (0.49; 1.21)^c^ |
| Olza J (2013) | Spain | Case-Control | 292/242 | rs2689248 | A | GoldenGate Assay | 0.79 (0.59; 1.07)^c^ |
| Olza J (2013) | Spain | Case-Control | 292/242 | rs17833492 | A | GoldenGate Assay | 0.88 (0.63; 1.22)^c^ |
| Olza J (2013) | Spain | Case-Control | 292/242 | rs7203521 | G | GoldenGate Assay | 0.93 (0.68; 1.28)^c^ |
| Olza J (2013) | Spain | Case-Control | 292/242 | rs10852521 | T | GoldenGate Assay | 0.67 (0.49; 0.90)^c^ |
| Olza J (2013) | Spain | Case-Control | 292/242 | rs8061518 | G | GoldenGate Assay | 0.56 (0.40; 0.78)^c^ |
| Olza J (2013) | Spain | Case-Control | 292/242 | rs17818902 | G | GoldenGate Assay | 1.19 (0.84; 1.67)^c^ |
| Olza J (2013) | Spain | Case-Control | 292/242 | rs10521303 | A | GoldenGate Assay | 0.84 (0.62; 1.15)^c^ |
| Olza J (2013) | Spain | Case-Control | 292/242 | rs7203181 | A | GoldenGate Assay | 0.88 (0.65; 1.19)^c^ |
| Olza J (2013) | Spain | Case-Control | 292/242 | rs7199716 | T | GoldenGate Assay | 0.91 (0.67; 1.24)^c^ |
| Olza J (2013) | Spain | Case-Control | 292/242 | rs12596638 | A | GoldenGate Assay | 0.99 (0.64; 1.53)^c^ |
| Meng XR (2014) | China | Case-Control | 1423/607 | rs62048402 | A | MassARRAY | **1.26 (1.02; 1.56)^c^** |

* Odds ratio (95% CI) under best genetic model, ^a^ Log-additive, ^b^ Allelic, ^c^ Additive
